# Tobacco use in older adults in Ghana: sociodemographic characteristics, health risks and subjective wellbeing

**DOI:** 10.1186/1471-2458-13-979

**Published:** 2013-10-20

**Authors:** Alfred E Yawson, Akosua Baddoo, Nana Ayegua Hagan-Seneadza, Benedict Calys-Tagoe, Sandra Hewlett, Phyllis Dako-Gyeke, George Mensah, Nadia Minicuci, Nirmala Naidoo, Somnath Chatterji, Paul Kowal, Richard Biritwum

**Affiliations:** 1 Department of Community Health, University of Ghana Medical School, College of Health Sciences, Room 46, P. O. Box 4236, Korle-Bu, Accra, Ghana; 2 Public Health Unit, Korle-Bu Teaching Hospital, Korle-Bu, Accra, Ghana; 3 University of Ghana Dental School, College of Health Sciences, University of Ghana, Korle-Bu, Accra, Ghana; 4 Department of Social and Behavioral Sciences, School of Public Health, College of Health Sciences, University of Ghana, Legon, Accra, Ghana; 5 National Council Research, Institute of Neuroscience, Padova, Italy; 6 World Health Organization, Multi-Country Studies unit, Geneva, Switzerland; 7 University of Newcastle Research Centre on Gender, Health and Aging, Newcastle, Australia

**Keywords:** Older adults, Tobacco use, Subjective wellbeing, Ghana, Middle income countries

## Abstract

**Background:**

Tobacco use over the life-course threatens to increase disease burden in older adulthood, including lower income countries like Ghana. This paper describes demographic, socioeconomic, health risks and life satisfaction indices related to tobacco use among older adults in Ghana.

**Methods:**

This work was based on the World Health Organization’s multi-country Study on global AGEing and adult health (SAGE), conducted in six countries including Ghana. Wave one of SAGE in Ghana was conducted in 2007-2008 as collaboration between WHO and the University of Ghana Medical School through the Department of Community Health. A nationally representative sample of 4305 older adults aged 50 years and above were interviewed. Associations between tobacco consumption and sociodemographic, socioeconomic, health risk and life satisfaction were evaluated using chi-square and odds ratio (OR). Logistic regression analyses, adjusted for age, sex and other variables, were conducted to determine predictors of tobacco consumption in older persons.

**Results:**

Overall prevalence of current daily smokers among older adults in Ghana was 7.6%. Tobacco use (i.e. ever used tobacco) was associated with older males, (AOR = 1.10, CI 1.05-1.15), older adults residing in rural locations (AOR = 1.37, CI 1.083-1.724), and older adults who used alcohol (AOR = 1.13, CI 0.230-2.418). Tobacco use was also associated (although not statistically significant per p-values) with increased self-reporting of angina, arthritis, asthma, chronic lung disease, depression, diabetes, hypertension, and stroke. Older adults who used tobacco and with increased health risks, tended to be without health insurance (AOR = 1.41, CI 1.111-1.787). Satisfaction with life and daily living was much lower for those who use tobacco. Regional differences existed in tobacco use; the three northern regions (Upper East, Northern and Upper West) had higher proportions of tobacco use among older adults in the country. Quitting tobacco use was higher in the 70+ years age group, in women, among urban residents and in those with at least secondary education. Quitting tobacco use also increased with increasing income levels.

**Conclusions:**

Tobacco use among older adults in Ghana was associated with older men living in rural locations, chronic ill-health and reduced life satisfaction. A high proportion of older adults have stopped using tobacco, demonstrating the possibilities for effective public health interventions. Health risk reduction strategies through targeted anti-smoking health campaigns, improvement in access to health and social protection (such as health insurance) will reduce health risks among older persons who use tobacco.

## Background

Current rising trends in tobacco use in low- and middle-income countries suggest this will increase the burden of non-communicable diseases in these countries [1]. Combined with demographic ageing in the same countries, the significance of chronic diseases among the ageing population and implications for quality of life of older adult population poses significant challenges [2].Tobacco use, diet rich in saturated fat and high in calories, excessive alcohol use and physical inactivity, are some of the modifiable risk factors contributing to cardiovascular diseases, cancer, chronic respiratory diseases and diabetes [3]. These non communicable disease (NCD) risks are strongly influenced by changing social and behavioural patterns and come at a high cost to health and economies, with lower income countries assuming an ever larger share of the economic burden of these factors and diseases [4].

Smoking has long been identified to be of prime importance in the cardiovascular health of adult Ghanaians [5] with more recent evidence suggesting it contributes more than 1% of total Disability Adjusted Life Years (DALYs) in Ghana [6]. The prevalence of tobacco use among older adults in Ghana is relatively low compared to some middle and high income countries [7-9]. Results from the 2003 Study on global AGEing and adult health (SAGE) in Ghana showed persistence of smoking into older ages; where 6.8% of the population 80-plus years were current daily smokers [10]. With even more recent evidence of increasing prevalence in younger populations, the impact and control of tobacco use was legislated through Public Health Bill 2011 in July 2012 [11]. This law is an important piece to help stem the impact of smoking on the population, with another key piece to address the risks posed by tobacco through evidence based research.

This research is based on the World Health Organization’s (WHO)2007/08 SAGE in Ghana and can be used as baseline data prior to implementation of the 2012 law. The goal is to describe the patterns of tobacco use among older adults (50 years and above) in Ghana by selected demographic, socioeconomic, health risk and life satisfaction indices and by location (administrative regions) to help identify high risk older adults in the population.

## Methods

### Introduction to SAGE

SAGE Wave 1 was undertaken in Ghana in a partnership between the University of Ghana’s Department of Community Health, the Ministry of Health and WHO, as part of a multi-country longitudinal study to complement existing ageing data sources to inform policy and programmes. A nationally representative sample was used for the survey(more details provided by Kowal and colleagues in a 2012 publication) [12]. Respondents 50 years and above were interviewed regarding their household characteristics, socio-demographic and work history, perceived health status, risk factors and preventive health behaviours, chronic conditions and health services coverage, health care utilization, subjective well-being and quality of life, and social cohesion. Field work and data entry were undertaken between May 2007 and June 2008. SAGE was approved by the World Health Organization's Ethical Review Board as well as a national approval in all six countries. Informed consent has been obtained from all study participants.

### Measures

#### Tobacco use

Lifetime tobacco use was assessed with the question ‘Have you ever smoked tobacco or used smokeless tobacco?’ Lifetime tobacco users were asked, ‘Do you currently use (smoke, sniff, or chew) any tobacco products such as cigarettes, cigars, pipes, chewing tobacco, or snuff?’ The pattern and quantity of tobacco consumption over the previous week was then collected. The mean number of tobacco products or cigarette equivalents consumed per day was designated as the mean daily tobacco consumption from the question ‘On average, how many of the following products (manufactured cigarettes, hand-rolled cigarettes, pipefuls of tobacco, cigars, and smokeless tobacco) do you smoke or use each day’.

#### Chronic diseases

SAGE gathered evidence on a selected range of chronic diseases typically more prevalent among older adults and that contribute to a large portion of non-communicable disease burden. In this analysis, data are presented for angina, asthma, chronic lung disease, depression, diabetes mellitus, hypertension, osteoarthritis and stroke. The prevalence rates for these chronic conditions were based on responses to the question “*Has a health care professional ever told you, you have…?"*.

#### Subjective wellbeing (SWB)

SWB or life satisfaction was assessed through a multi-dimensional scale, including a question about satisfaction with life overall. The response to this last question was used as the single item measure for SWB in this analysis. Three categories of SWB was then used, satisfied (for responses as very satisfied and satisfied), indifferent (for neither satisfied nor dissatisfied) and not satisfied (for dissatisfied and very dissatisfied).

#### Sociodemographic characteristics

Including age, sex, highest level of education, marital status and socioeconomic status were collected. Wealth or income quintiles were derived from the household ownership of durable goods, dwelling characteristics and access to services (improved water, sanitation and cooking fuel) for a total of 21 assets. Wealth levels were generated through a multi-step process, where asset ownership was converted to an asset ladder, Bayesian post-estimation method used to generate raw continuous income estimates, and then income transformed into quintiles [13].

#### Health insurance

Ghana operates a social health insurance policy, the National Health Insurance Scheme, introduced in 2003 and became operational in most public and private health facilities in 2005 [14]. There are a few private, voluntary and mutual health insurance schemes operated by health and corporate organizations in the urban centres. The health insurance status of respondents were determined as Yes (mandatory, voluntary or both) or No (no insurance).

#### Data analysis

Associations between tobacco use and sociodemographic, socioeconomic, health risk and life satisfaction indices were evaluated using chi-square and odds ratio (OR). Demographic and socio-economic variables used included age, region of residence, location (urban/ rural), educational level, marital status, and income levels.

Logistic regression analysis was conducted to determine predictors of tobacco use in the older persons. The logistic regression model (using adjusted odds ratio [AOR] and p-values at 95% confidence level) had use or life-time abstinence from tobacco in the older adult as binary dependent variable and independent variables used included age, sex, location (urban/rural), educational level, marital status, income quintile, health insurance status, self-reported chronic conditions, alcohol use and subjective wellbeing.

A nationally representative sample of 4305 older adults aged 50 years and above were interviewed and data used for the analysis was weighted and age-standardized. Analysis was conducted using SPSS version 21.

## Results

### Patterns of tobacco use in the older adults

Overall, 7.6% were current daily smokers, 2.6% were current smokers, but not daily while three-quarters of older Ghanaians had never smoked (75.5%). The proportion of respondents who were current daily smokers was higher among men (M:F ratio = 3.1), among rural residents, and increased with age (from 6.8% in 50-59 years to 8.2% in those 70 years and above). Respondents with secondary school (or equivalent) completed (11.9%) and those without formal education clearly showed higher proportions of current smokers (10.2%), compared to those with college/university or postgraduate education (1.6%). The proportion of current daily smokers decreased dramatically with income. Compared to the currently married/cohabiting, the widowed had lower proportions of current daily smokers (5.8% compared to 9.0%).

Amongst older women, 92.0% had never smoked, compared to 64.4% of older men (M:F ratio of 0.7). Higher proportions of respondents who had never smoked were urban residents, younger (50-59 years), and with higher education. Older adults who have previously smoked but are currently not smoking (shown as not current smoker in Table 1) was highest in the 70+ age group, was higher in women compared to men, was higher among urban residents and those with at least secondary education. Older adults who have quit smoking increased with income levels, was lowest (11.8% among the Q1 group) and highest (16.8% among the Q5 group).

**Table 1:** Prevalence of smoking and average daily tobacco consumption in older adults, by selected characteristics

**Characteristics**	**Tobacco use**				**Mean daily tobacco use (cigarette equivalent)**	**N (4252)**
	**Current daily smoker (%)**	**Smoker, not daily (%)**	**Not current smoker (%)**	**Never smoker (%)**		
**Age group**						
50–59	6.8	2.9	14.0	76.4	5.7	1 694
60–69	7.6	3.0	13.1	76.3	5.1	1 172
70+	8.7	2.0	15.5	73.8	4.8	1 386
**Sex**						
Male	11.3	3.7	24.6	60.4	5.4	2 090
Female	3.7	1.4	29.0	92.0	4.4	2 160
M : F Ratio	3.1	2.6	0.8	0.7	1.2	
**Residence**						
Urban	4.1	2.2	15.6	78.2	4.8	1 753
Rural	10.2	2.9	13.3	73.7	5.3	2 497
**Education**						
No education	10.2	1.8	10.7	77.2	5.0	2 310
Primary completed or less	4.3	2.4	16.1	77.2	4.9	886
Secondary education(or equivalent) completed	11.9	7.5	43.2	13.3	15.6	882
College/University/ Postgraduate	1.6	0.9	31.8	16.7	3.5	149
**Marital status**						
Never married	12.2	4.3	21.6	61.9	3.3	61
Currently married/ Cohabited	9.0	3.0	18.8	69.2	5.5	2441
Separated/divorced	4.9	3.3	12.3	79.6	5.2	547
Widowed	5.8	1.3	4.8	88.1	4.1	1 177
**Income quintile**						
Q1	16	3.2	11.8	69.0	4.4	790
Q2	9.1	3.1	13.3	74.5	5.1	813
Q3	8.0	2.1	14.5	75.4	6.2	865
Q4	4.8	2.3	14.2	78.7	5.2	862
Q5	1.8	2.5	16.8	78.9	7.4	916
**Total respondents (%)**	325 (7.6)	111(2.6)	605 (14.2)	3 211(75.5)	325 (7.6)	4252

The highest mean daily tobacco use decreased with age, however, the habit of tobacco use persisted into older age (70-plus age group still recorded a mean daily tobacco use of 4.8). Mean daily use of tobacco was higher for men (5.4) than women (4.4) and higher among rural residents (5.3) than urban residents (4.3). Respondents with at least secondary school (or equivalent) had the highest mean daily tobacco use (15.6) compared to those without formal education (5.0). The mean daily tobacco use was relatively higher among the currently married than the widowed.

### Regional distribution of tobacco use in the older adults

Overall, the national prevalence of current daily smoking among older Ghanaians was 7.6%. Table 2 shows the relative percentage contribution of each region to the total number of older adults who use tobacco and indicates that, the three northern regions have the highest relative percentages of current daily smoking; 31.2% in Upper East, 22.5% in Northern and 7.9% in Upper West. The Ashanti and Greater Accra regions had the lowest relative percentage of current daily smoking, (2.6% and 3.1% respectively). The Brong Ahafo (being a transitional regional belt between the northern regions and the southern regions) had proportion of current daily smokers which was intermediate (7.2%).

**Table 2:** Patterns of overall tobacco use in older adults, by region

**Characteristics**	**Tobacco use (%)**			**N**
	**Current daily smoker**	**Smoker, not daily**	**Not current smoker**	
Ashanti	2.6	15.3	18.7	670
Brong Ahafo	7.2	13.6	11.2	409
Central	5.8	8.5	9.8	452
Eastern	6.2	8.5	13.1	557
Greater Accra	3.1	13.6	12.8	493
Northern	22.5	7.6	6.3	385
Upper East	31.2	4.2	3.1	260
Upper West	7.9	6.8	1.7	132
Volta	5.8	8.5	9.1	411
Western	7.7	13.6	14.2	511
Total	100	100	100	4280

The Ashanti, Western and Greater Accra regions had relatively higher proportions of older persons who had smoked previously but were currently not smoking compared to the three northern regions. For example the Ashanti region had 18.7% of older persons who had smoked previously but were currently not smoking compared to 3.3% in the Upper East region (the region with the highest proportion of current daily smokers).

### Demographic, socioeconomic, health risks and life satisfaction indices associated with tobacco use in older adults in Ghana

Results from logistic regression shown in Table 3 indicate that demographic, socioeconomic, chronic ill-health and life satisfaction indices have significant association with tobacco use in the older adults. Older males had a slightly higher risk of tobacco use (AOR = 1.10, CI 1.05-1.15), as well as older adults residing in rural location (AOR = 1.37, CI 1.083-1.724). Older adults without health insurance also had increased risk of tobacco use (AOR = 1.41, CI 1.111-1.787). Satisfaction with life was much lower for the older adults who use tobacco. There was increased self-reporting of dissatisfaction with life more for the older adults who use tobacco (AOR = 3.26, CI 1.190-5.928).

**Table 3:** Predictors of tobacco use in older adults in Ghana

**Characteristic**	**Predictive factor**	**P-value**	**Odds ratio**	**95% confidence interval**
**Tobacco use**	**Sex**			
	Female	*Ref*		
	Male	0.001	1.100	1.046-1.148
	**Location**			
	Urban	*Ref*		
	Rural	0.009	1.366	1.083-1.724
	**Age group**			
	50-59	*Ref*		
	60-69	0.992	1.002	0.723-1.389
	70 and above	0.348	0.849	0.602-1.195
	**Educational level**			
	No formal education	*Ref*		
	With education	0.919	1.012	0.798-1.285
	**Marital status**			
	Never married	*Ref*		
	Living with partner (currently married and cohabiting)	0.088	0.277	0.063-1.211
	Living without partner (separated/divorced and widowed)	0.779	0.875	0.343-2.231
	**Income level**			
	Lower income (Q1,Q2 and Q3)	*Ref*		
	Higher income (Q4 and Q5)	0.460	1.408	0.567-3.495
	**Health insurance status**			
	Insured	*Ref*		
	Uninsured	0.005	1.409	1.111-1.787
	**Lifetime alcohol use**			
	Never	*Ref*		
	Ever	0.001	1.310	0.230-2.418
	**Self-reported angina**			
	No	*Ref*		
	Yes	0.323	1.532	0.658-3.566
	**Self-reported arthritis**			
	No	*Ref*		
	Yes	0.009	1.625	0.440-2.889
	**Self-reported asthma**			
	No	*Ref*		
	Yes	0.805	1.075	0.607-1.903
	**Self-reported chronic lung disease**			
	No	*Ref*		
	Yes	0.139	1.387	0.298-2.272
	**Self-reported depression**			
	No	*Ref*		
	Yes	0.291	1.613	0.247-2.520
	**Self-reported diabetes**			
	No	*Ref*		
	Yes	0.659	1.894	0.542-2.473
	**Self-reported hypertension**			
	No	*Ref*		
	Yes	0.809	1.024	0.731-1.435
	**Self-reported stroke**			
	No	*Ref*		
	Yes	0.914	1.035	0.555-1.930
	**Level of subjective well being/ life satisfaction**			
	Satisfied	*Ref*		
	Indifferent	0.006	4.115	1.505-8.252
	Not satisfied	0.022	3.259	1.190-5.928

Although not statistically significant (per p-values in Table 3), there was increased associations (per adjusted odds ratios in Table 3) for self-reporting of chronic diseases among older adults who use tobacco for hypertension, stroke, diabetes, depression, chronic lung disease, asthma, arthritis and angina. Increased association with arthritis among older adults who use tobacco was statistically significant.

## Discussion

Tobacco use has significant impact on mortality and morbidity from non-communicable diseases, based on recent estimates of mortality rates in the 50-54 age group of 34 per 100,000 population to 158 per 100,000 population in the 80+ age group and contributing up to 1% of DALYs in Ghana according to the 2010 Global Burden of Disease study [6]; however, risk modification is possible through effective primary prevention and health promotion efforts [2,15].

Prevalence of current daily smokers in older Ghanaians was 7.6% and was higher among men (with M: F ratio of 3.1). This figure compares favourably to “ever smoking” rate but is much higher than current rate found in 2009 by Owusu-Dabo and colleagues in Ghana [16]. The reported prevalence of smoking was relatively higher for both men and women in SAGE Wave 1, than in SAGE Wave 0 in 2003 among the population aged 50+ years [10]. In SAGE Wave 1, prevalence of current daily was 11.3% in males and 3.7% in females compare to the SAGE Wave 0 where it was 7.5% in males and 0.7% in females [10]. The prevalence of current daily smokers was also higher among rural residents (probably due to the use of smokeless tobacco among the rural Ghanaian population). The 2008 Ghana Demographic and Health Survey report also indicated higher use of tobacco among males than females and more in rural than urban locations [9].

Prevalence of current daily smokers increased with age and decreased dramatically as income increased. Respondents without formal education clearly showed higher proportions of current smokers (10%), compared to those with College/University or Postgraduate education (2%). The older persons who were never married had the highest prevalence of current daily smokers (12%), while the widowed had lower proportions (6%) than those currently married/ cohabiting (9%). The mean daily tobacco use (number of cigarette/cigarette equivalent) generally followed the socio-demographic patterns described for the current daily smokers. A recent study by Blazer and Wu,2012 among older Americans showed similar sociodemographic correlates to tobacco use; prevalence of tobacco use was higher among males, those with less than high school education, those who were never married and among the unemployed and lower income groups [17].

There were clear regional differences in the distribution of tobacco use in the older adults. The three northern regions had the highest proportion of current daily smoking; 31.2% in Upper East, 22.5% in Northern and 7.9% in Upper West. The two most populous and most developed regions (Greater Accra and Ashanti) had the lowest proportion of current daily smoking, (2.6%) in Ashanti and (3.1%) in Greater Accra. These regional differences are in conformity with findings in a 2012 analysis of the Ghana Demographic and Health Survey by Rijo and colleagues who found that tobacco use was significantly higher among those living in poverty stricken regions [18]. These differences in tobacco use based on population sizes and level of development in different localities, are also similar to the study by Blazer and Wu. They found that, older adults residing in non-metropolitan areas had higher prevalence and those in large-metropolitan areas had the lowest prevalence [17].

The increase prevalence of tobacco use with age poses major health challenges. Like cigarette smoking, smokeless tobacco use, cigar, and pipe smoking all contribute to addiction, cancers, heart disease, and respiratory conditions [7,8,17-20]. Due to related changes in health status [17,21,22], tobacco use affects older adults by exacerbating existing diseases, causing poorer physical functioning, prompting costly treatment use, and increasing mortality [23,24]. Cessation of tobacco use is critical for older adults because it reduces functional impairments and mortality associated with respiratory and cardiovascular diseases [17,24].

The prevalence of tobacco use among older adult Ghanaians is relatively low compared to many middle- and higher- income countries [7-9]. However, the impact of tobacco use on older Ghanaians is sufficient to warrant national attention. These are mostly aged men residing in rural communities, with little or no education and in the lower income group for whom access to health care is a challenge. Low education, inadequate information, limited access to jobs or income and concomitant inadequate health screening, as well as age-related co-morbid conditions might be more prevalent in this identifiable older adult group.

Results from the logistic regression indicate that demographic, socioeconomic, chronic ill-health and life satisfaction indices have significant association with tobacco use in the older adults. Older males and older adults residing in rural locations had a higher association with tobacco use. The combined use of tobacco and alcohol in older adult pose significant health challenges [3,17,19]. Importantly, SAGE can be used to further document impact and inform public health measures and also as a monitoring mechanism for the 2011 tobacco legislation [11] with SAGE Ghana Wave 2 planned for 2013/14 and Wave 3 two years after this.

Associations were found between self-reported chronic diseases among older adults who use tobacco. Interestingly, older adults who use tobacco tended to be without health insurance and this is similar to findings from the 2008 Ghana Demographic and Health Survey [9]. This observation is of major public health policy implications i.e. the older adults in Ghana who have increased risk for chronic health conditions as a result of tobacco use, tend not to be health insured. Add to this the reduced financial capacity in lower economic rungs of the population and poor coverage of social protection system (pension and health insurance), an active preventive health approach directed at older adults should be operationalized as part of Priority Direction II in the 2010 National Ageing Policy.

Self-reported satisfaction with life was much lower for the older adults who use tobacco. Dissatisfaction with activities of daily living or low social wellbeing has implications for the health of the individual and the social well being of dependants of these older adults.

The benefit of decreasing the risk for stroke for example among older adults appears particularly evident among non-heavy smokers [25]. Unfortunately, research has suggested a low rate of smoking cessation among older adults [26]. Interestingly, findings on older adults who have quit smoking revealed that the proportion of those who have quit smoking was higher in the 70+ age group, in women, among urban residents, those with at least secondary education and increased with income levels. In addition the more urbanized regions, Greater Accra, Ashanti and Western regions had higher proportions of older persons who have quit smoking compared to the least urbanized three northern regions. This may probably be due to increased exposure and awareness of harmful effects of tobacco in older adults living in these most urbanized regions of the country [18,27]. Also older persons may become more aware of the harmful effects of smoking, through life experiences by age, through formal education, and increased exposure to public education and media (in the urban areas). In our setting, other risk reduction measures such as improvement in access to health and social services might engender more health and social benefits when the most at risk population groups are targeted in national health risk reduction policies and programmes [28].

The possibility that self-reported tobacco use data underreports the actual prevalence due to inhibitions in admitting to use is a limitation of this study, and may well result in higher estimates than obtained from the survey. Also the self-report of tobacco use as a health variable should be interpreted with caution due to potential measurement errors and also, the questions asked on the quantity or intensity of tobacco use depended on memory, therefore, there is the possibility of recall bias. In the survey however, the questions referred to very short time periods to minimize the potential recall bias [12,29].

## Conclusion

The prevalence of tobacco use among older adult Ghanaians is relatively low compared to many middle- and higher- income countries. However, the impact of tobacco use on older Ghanaians is sufficient to warrant national attention. The survey data demonstrated that older men, rural dwellers, those without national health insurance and older adults with other co-morbid health conditions may be at increased risk.

We recommend;

● risk modification through effective primary prevention and health promotion efforts

● health risk reduction strategies, including public health campaigns linked to the 2011 legislation on ban of tobacco use in public places

● improvement in access to health and social protection such as health insurance for older adults

## Competing interests

The authors declare no competing interest. The views expressed in this paper are those of the authors. No official endorsement by the World Health Organization or Ministry of Health of Ghana/ Ghana Health Service is intended or should be inferred.

## Authors' contributions

AEY, ANB and NAH-S developed the concept, AEY, GM, NM, NN N^9^, SC, PK and RBB are members of the WHO Multi-country SAGE Study Team involved in the conduct and analysis of the SAGE survey in Ghana. ANB, SH, BNLC-T, PD-G, NAH-S and AEY contributed to the writing and reviewing of the various sections of the manuscript. All the authors reviewed the final version of the manuscript before submission. All authors read and approved the final manuscript.

## Authors’ information

Yawson AE, Mensah G, MinicuciN and Biritwum RB are members of the WHO Multi-country SAGE Team who conducted the SAGE Survey in Ghana. Naidoo N, Chatterji S and Kowal P are members of the WHO Multi-country SAGE Team and coordinators of the multi-country study at the WHO Headquarters in Geneva.

AN Baddoo, BNL Calys-Tagoe, and NA Hagan-Seneadza are Specialist Public Health Physicians, Dako-Gyeke P is a Social scientist and University lecturer. Hewlett S is a University lecturer and specialist Dental surgeon.

## Pre-publication history

The pre-publication history for this paper can be accessed here:

http://www.biomedcentral.com/1471-2458/13/979/prepub

## References

[B1] The PLOS Editors M Addressing global disparities in the burden of non communicable diseases: call for papers PLoS Med 2012 13 12 e1001360 10.1371/ journal.pmed.1001360

[B2] United Nations Population Division, DESA World Population Prospects 2011 New York: United Nations: The 2010 Revision

[B3] World Health Organization The World Health Report Reducing risks, promoting healthy life 2002 Geneva: World Health Organization

[B4] Bloom DE Cafiero ET Jane Llopis E Abrahams Gessel S Bloom LR Fathima S The Global Economic Burden of Non communicable Diseases 2011 Geneva: World Economic Forum www3.weforum.org/docs/WEF_Harvard_HE_GlobalEconomicBurdenNonCommunicableDiseases_2011.pdf

[B5] Pole D Ikeme AC Pobee JO Larbi E Williams H Blankson J The Mamprobi Survey–a screening survey for cardiovascular disease and risk factors in Africa: methodology and validity Bull World Health Organ 1979 13 81 87 311714 PMC2395756

[B6] Global Burden of Disease Study 2010 2012 http://www.healthmetricsandevaluation.org/sites/default/files/country-profiles/GBD%20Country%20Report%20-%20Ghana.pdf

[B7] National Cancer Institute (NCI) Smokeless tobacco and cancer [online] 2013 from http://www.cancer.-gov/cancertopics/factsheet/Tobacco/smokeless, 2011

[B8] National Cancer Institute (NCI) (2011b) Cigar smoking and cancer 2011 from http://www.cancer.gov/cancertopics/factsheet/Tobacco/cigars

[B9] Ghana Statistical Service (GSS) Ghana Health Service (GHS), and ICF Macro. Ghana Demographic and Health Survey 2008 2009 Accra, Ghana: GSS, GHS, and ICF Macro

[B10] Department of Community Health, University of Ghana Medical School Ghana National Report on World Health Survey (WHS) 2003 Published by the Department of Community Health, University of Ghana Medical School in collaboration with WHO http://www.who.int/healthinfo/survey/whsgha-ghana.pdf

[B11] Public Health Bill 2011 of the Republic of Ghana 2012 Accra, Ghana: Published by the Ghana Assembly Press http://www.africatobaccocontrol.org/en/index.php/media-center/latest-news/272-ghana-parliament-passes-the-public-health-bill-with-tobacco-control-measures

[B12] Kowal P Chatterji S Naidoo N Biritwum R Wu F Lopez Ridaura R Maximova T Arokiasamy P Phaswana-Mafuya N Williams S Snodgrass JJ Minicuci N D'Este C Peltzer K Boerma JT the SAGE Collaborators Data Resource Profile: The World Health Organization Study on global AGEing and adult health (SAGE) Int J Epidemiol 2012 1 11 doi:10.1093/ije/dys210 23283715 10.1093/ije/dys210 PMC3535754

[B13] Biritwum RB Mensah G Minicuci N Yawson AE Naidoo N Chatterji S Kowal P Study on global AGEing and adult health in Ghana: Methodology and household characteristics in Wave 1 Glob Health Action 2013 13 20096 http://dx.doi.org/10.3402/gha.v6i0.20096 23759325 10.3402/gha.v6i0.20096 PMC3681208

[B14] National Health Insurance Authority of Ghana Annual Report of the National Health Insurance Scheme of Ghana (2010) 2011

[B15] World Health Organization 2013 http://blogs.bmj.com/tc/2013/07/11/new-who-report-more-tobacco-advertising-bans-smoke-free-spaces-save-millions-of-lives/?q=w_tc_blog_sidetab

[B16] Owusu-Dabo E Lewis S McNeill A Gilmore A Britton J Smoking uptake and prevalence in Ghana Tob Control 2009 13 365 370 10.1136/tc.2009.030635 10.1136/tc.2009.030635 19581276 PMC2745559

[B17] Blazer DG Wu L Patterns of tobacco use and tobacco-related psychiatric morbidity and substance use among middle-aged and older adults in the United States Ment Health Ment Health 2012 13 3 296 304 10.1080/13607863.2011.615739 PMC3323715 22292514

[B18] John RM Mamudu HM Liber AC Socioeconomic implications of tobacco use in Ghana Nicotine Tob Res 2012 13 10 1205 12 10.1093/ntr/nts013 10.1093/ntr/nts013 22387993

[B19] Henley SJ Thun MJ Chao A Calle EE Association between exclusive pipe smoking and mortality from cancer and other diseases J Natl Cancer Inst 2004 13 853 861 10.1093/jnci/djh144 15173269

[B20] Wipfli H Samet JM Global economic and health benefits of tobacco control: Part 1 Clinical Pharmacol Ther 2009 13 263 271 10.1038/clpt.2009.93 19536067

[B21] Gibson P Mcdonald V Marks G Asthma in older adults Lancet 2010 13 803 813 10.1016/S0140-6736(10)61087-2 20816547

[B22] Gooneratne N Patel N Cocoran A Chronic obstructive pulmonary disease diagnosis and management in older adults J Am Geriatr Soc 2010 13 1153 1162 10.1111/j.1532-5415.2010.02875.x 20936735

[B23] Tice JA Kanaya A Hue T Rubin S Buist DSM LaCroix A Bauer DC Risk factors for mortality in middle-aged women Arch Intern Med 2006 13 2469 2477 10.1001/archinte.166.22.2469 17159012

[B24] Lacroix A Omenn G Older adults and smoking Clin Geriatr Med 1992 13 69 87 1576581

[B25] Wannamethee SG Shaper AG Whincup PH Walker M Smoking cessation and the risk of stroke in middle-aged men JAMA 1995 13 155 160 10.1001/jama.1995.03530020073035 7596004

[B26] Salive M Cornoni-Huntley J Lacroix A Ostfeld AM Wallace RB Hennekens CH Public health briefs Am J Public Health 1992 13 1268 1271 10.2105/AJPH.82.9.1268 1503170 PMC1694340

[B27] Ghana Statistical Service Ghana Living Standards Survey Report of the Fifth Round (GLSS 5). Ghana Statistical Service 2008 Published by Ghana Statistical Service

[B28] Doku D Raisamo S Wiium N The role of tobacco promoting and restraining factors in smoking intentions among Ghanaian youth BMC Public Health 2012 13 662 10.1186/1471-2458-12-662 10.1186/1471-2458-12-662 22894679 PMC3490846

[B29] Peltzer K Phaswana Mafuya N Fruit and vegetable intake and associated factors in older adults in South Africa Glob Health Action 2012 13 18668 http://dx.doi.org/10.3402/gha.v5i0.18668 10.3402/gha.v5i0.18668 PMC3511777 23195518

